# Lowering blood pressure in primary care in Vienna (LOW-BP-VIENNA): A cluster-randomized trial

**DOI:** 10.1007/s00508-018-1374-4

**Published:** 2018-08-15

**Authors:** Miklos Rohla, Maximilian Tscharre, Kurt Huber, Thomas W. Weiss

**Affiliations:** 1 3rd Medical Department, Cardiology and Intensive Care Medicine, Wilhelminenhospital, Vienna, Austria; 2 Institute for Cardiometabolic Diseases, Karl Landsteiner Society, St. Pölten, Austria; 3 Medical Faculty, Sigmund Freud University, Vienna, Austria

**Keywords:** Arterial hypertension, Hypertension control, Disease management programs, Single pill combination drugs, Ambulatory blood pressure measurement

## Abstract

**Background:**

In Austria only 41% of patients with treated hypertension (HTN) have their blood pressure (BP) controlled. This study investigated a strategy to improve BP control in primary care.

**Methods:**

General practitioners (GPs) were randomized to interventional care vs. standard care and included patients with uncontrolled office BP > 140/90 mm Hg. In interventional care, antihypertensive therapy was up-titrated using a single pill combination (olmesartan, amlodipine and/or hydrochlorothiazde) in 4‑week intervals. In standard care, physicians were encouraged to treat according to the 2013 European Society of Cardiology guidelines for the management of arterial hypertension. The primary endpoint was the proportion of patients with controlled office BP < 140/90 mm Hg at 6 months. The main secondary endpoint was the improvement in 24 h ambulatory BP (ABPM, Clinicaltrials.gov NCT02377661).

**Results:**

Between 2015–2017, 20 GPs contributed to patient recruitment. The trial was discontinued due to slow recruitment after inclusion of 139 eligible patients, 54 of whom were included in the interventional group. A significantly larger proportion of patients in interventional vs. standard care achieved the office BP target (67% ± 26% vs. 39% ± 29%, respectively, mean difference −27.9%, 95% confidence interval CI −54.0%; −1.7%, *p* = 0.038). The proportion of patients with controlled 24 h ABPM (<130/80 mm Hg) was similar between groups (49% ± 33% vs. 40% ± 34%, respectively, mean difference −8.8%, 95% CI −40.7%; 23.1%, *p* = 0.57). At baseline, pretreated patients received an average of 1.5 ± 0.8 vs. 1.7 ± 0.9 antihypertensive prescriptions. At 6 months, the respective BP reductions were achieved with 1.2 ± 0.5 prescriptions in interventional vs. 2.0 ± 1.0 in standard care (*p* < 0.01).

**Conclusion:**

In both groups statistically and clinically significant BP reductions were observed after 6 months. In the interventional care group, a larger proportion of patients achieved the office BP target compared to standard care. The 24 h ambulatory blood pressure levels were controlled in 44% of patients at 6 months, without significant differences between groups. The respective BP reductions were achieved with a significantly lower medication burden in interventional care.

**Electronic supplementary material:**

The online version of this article (10.1007/s00508-018-1374-4) contains supplementary material, which is available to authorized users.

## Introduction

In Europe only 30–50% of diagnosed and treated patients with arterial hypertension (HTN) have their blood pressure (BP) controlled [1, 2]. The asymptomatic nature of the condition combined with frequent adverse effects of antihypertensive drugs lead to therapy discontinuation in up to 50% of patients within 1 year of treatment [3]. Another barrier to adequate BP control is physician’s inertia, i.e. the lack of therapy intensification in cases of insufficient BP. This group recently performed a cross-sectional study in Austria, showing that only 41% out of 4303 predominantly adherent, diagnosed and treated patients had their BP controlled. These patients received an average of 1.8 different antihypertensive drugs, suggesting sufficient room for therapy intensification, rather than treatment resistance [4]. Considering that a population-based BP reduction as little as 2 mm Hg would be associated with a 10% decrease in stroke-related deaths, disease management programs seem worthwhile for most European countries [5]. The study investigated a strategy to improve BP control in primary care, comparing standard treatment to a prespecified titration regimen with single pill combinations (SPCs).

## Methods

### Trial design

The lowering blood pressure in primary cin Vienna (LOW-BP-VIENNA) trial was a prospective cluster-randomized controlled multi-center trial designed to compare standard treatment for HTN vs. interventional care with a prespecified titration regimen using a SPC. General practitioners (GPs) or resident specialists for internal medicine were enrolled via a written invitation or telephone interview. All participating study sites were required to have an active contract with the public health insurance. Study sites were allocated to either standard or interventional care at the beginning of the trial in a 1:1 fashion using a random sequence generator. The study was approved by the national regulatory authority and ethics committee. All participants gave written informed consent. The trial was registered with clinicaltrials.gov (NCT02377661).

### Participants

The study included patients aged 18–80 years with a systolic/diastolic office BP of ≥140/≥90 mm Hg. Patients with a malignant disease and a life expectancy <6 months, contraindications or allergies to olmesartan, amlodipine or hydrochlorothiazide (interventional arm only), previously diagnosed chronic kidney disease grade IV or V, recent myocardial infarction or stroke within the preceding 3 months, participation in another clinical trial and women of childbearing potential or currently breastfeeding were excluded from the trial.

### Procedures

Office BP was determined according to the 2013 European Society of Hypertension and European Society of Cardiology (ESH/ESC) guidelines on the management of arterial hypertension using semi-automated oscillometric devices [6]. At least one reading on each arm was obtained and as qualifying reading, the highest BP was used unless an outlier was suspected. All eligible patients were scheduled to receive a 24 h ambulatory blood pressure measurement (ABPM, Mobil-O-Graph PWA, I.E.M., Stolberg, Germany) at enrolment (before modification of antihypertensive therapy) and after 6 months of follow-up. Physicians could exclude patients in whom therapy intensification might not be indicated based on APBM readings at their own discretion, particularly in the case of white coat hypertension (elevated office BP levels, normotensive ABPM levels) or masked hypertension (normotensive office BP levels, elevated ABPM levels). In total, 4 patients were discontinued due to white coat hypertension and 2 patients were discontinued due to masked hypertension at the physician’s discretion. Physicians randomized to standard of care were encouraged to intensify antihypertensive therapy in line with recommendations from the 2013 ESH/ESC guidelines for the management of arterial hypertension [6]. The study protocol suggested monthly follow-up visits until 6 months of follow-up.

**Fig. 1:**
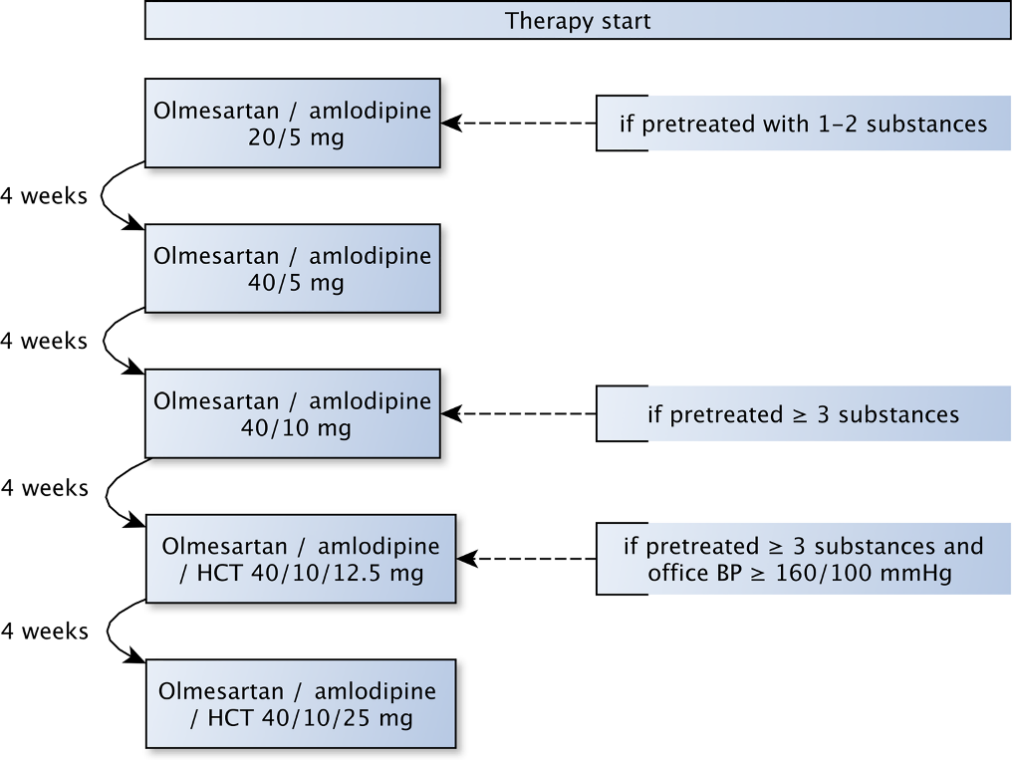
Prespecified titration regimen with the single pill combination drug in the interventional care group. *BP* blood pressure, *HCT* hydrochlorothiazide

In the interventional care arm, previous antihypertensive medication was discontinued (except for beta-blockers for rate control in atrial fibrillation and following myocardial infarction) and replaced by a SPC including olmesartan, amlodipine and/or hydrochlorothiazide (HCT). Initial dosing recommendations of the SPC and dose escalation steps were in analogy to the BP-CRUSH trial and are presented in Fig. 1 [7]. Follow-up visits were scheduled monthly until 6 months of follow-up. In the case of normotensive systolic/diastolic office BP levels (<140/<90 mm Hg) during any time of follow-up, the dose was maintained, but could be increased in cases of uncontrolled BP at any of the subsequent follow-ups. In cases of uncontrolled BP despite escalation to the maximum dose, other antihypertensive agents could be added at the physician’s discretion.

### Outcome measures

The primary endpoint was the proportion of patients achieving the target office BP of <140/90 mm Hg at 6 months of follow-up at the cluster level. Main secondary outcomes included the achievement of average systolic 24 h ABPM <130 mm Hg, average diastolic 24 h ABPM <80 mm Hg and achievement of average daytime (135/85 mm Hg) and nighttime (120/70 mm Hg) BP levels at 6 months of follow-up at the cluster-level. Additionally, office BP and ambulatory BP data were compared between groups at the patient level.

### Sample size

A between-group difference for the proportion of patients achieving the target office BP of 10% (65% vs. 55% for patients in interventional vs. standard care) was estimated [8]. With an alpha level of 0.05 and a power of 80%, the sample size needed to show statistical significance was 375 per treatment arm [9]. Taking into account a 12% lost to follow-up rate, a total sample size of 840 was estimated.

### Statistical analysis

Discrete characteristics are expressed as frequency counts and percentages, and differences between groups were determined by the χ^2^-test. Continuous, normally distributed variables are expressed as means with standard deviations (SD), unless otherwise specified. Differences were examined using the Student’s t‑test or the Mann-Whitney test, where appropriate. The level of significance used for all tests was a two-sided *p* value of 0.05. Because the intervention was implemented on a practice level, a cluster-randomized design was adopted. Accordingly, the proportion of patients achieving target BP levels was analyzed at the cluster level rather than the individual patient level using a 2-sample *t-*test [8]. To account for differences in the size of the clusters, the primary and secondary endpoints were additionally analyzed using a weighted *t-*test [10]. Patient level data (office BP and ABPM) were compared between groups using a 2-sample *t-*test.

## Results

### Study sites and patients

**Fig. 2:**
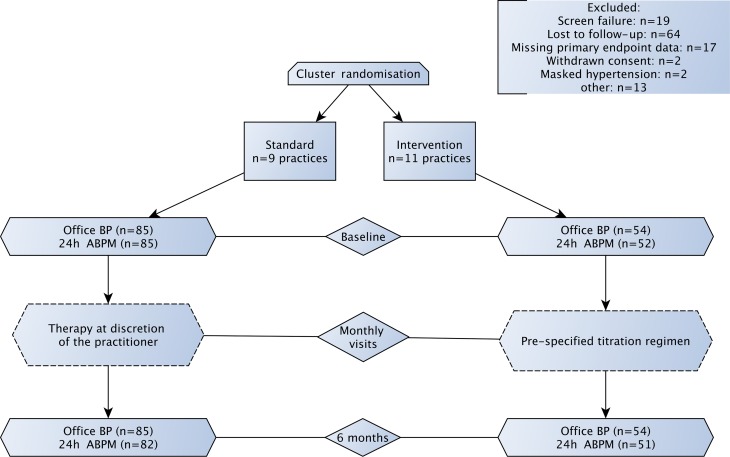
Flow diagram showing the study design, the number of sites and the number of participants in each trial arm. *BP* blood pressure, *ABPM* ambulatory blood pressure measurement

Initially, 29 GPs and 4 specialists for internal medicine were randomized, of whom 20 contributed to patient enrolment. The trial was discontinued due to slow recruitment after inclusion of 256 patients between March 2015 and Mai 2017. In total, 117 patients were excluded from the final analysis, of whom 19 did not fulfil the inclusion criteria, 64 were lost to follow-up, 17 had missing primary endpoint data and 17 for other reasons (Fig. 2).

The outcomes of 139 eligible participants with available data for the primary endpoint (*n* = 85 standard, *n* = 54 intervention) are reported. On average, 7 ± 6 patients were included per cluster (minimum 1, maximum 20).

Patients mean age was 59 ± 11 years, 53% were female and 80% were previously treated for HTN. Baseline demographics were well-matched between groups (Table 1). Patients in interventional care had significantly higher systolic office BP levels (165 ± 17 vs. 159 ± 18 mm Hg, respectively, *p* = 0.01); however, baseline ABPM values were similar between groups (Table 1).

**Table 1:** Baseline demographic and clinical data for the standard and interventional treatment groups

		Standard	Intervention	*p*-value
Age (years)		58.0 ± 12.0	59.0 ± 9.0	0.97
BMI		31.9 ± 14.5	29.6 ± 6.1	0.17
Heart rate		75.3 ± 14.5	75.6 ± 12.5	0.73
Office SBP at baseline		158.8 ± 18.0	164.8 ± 17.1	0.01
Office DBP at baseline		94.6 ± 9.1	95.0 ± 12.6	0.84
24 h SBP at baseline		139.9 ± 13.9	141.9 ± 14.0	0.31
24 h DBP at baseline		85.9 ± 9.8	86.0 ± 9.4	0.86
Daytime SBP at baseline		142.8 ± 14.9	143.8 ± 13.7	0.51
Daytime DBP at baseline		88.5 ± 11.0	88.0 ± 10.1	0.89
Nighttime SBP at baseline		132.0 ± 15.2	136.1 ± 18.3	0.15
Nighttime DBP at baseline		79.0 ± 10.3	80.4 ± 11.8	0.66
Female gender		57.60%	44.40%	0.13
Marital status	Single	11.80%	16.70%	0.48
	Married or partnership	60.00%	64.80%	–
	Divorced	15.30%	7.40%	–
	Widowed	12.90%	11.10%	–
Employment	Employed	35.30%	36.50%	0.80
	Retired	50.60%	46.20%	–
	Unemployed	10.60%	15.40%	–
	Self-employed	3.50%	1.90%	–
Highest level of education	Compulsory education	27.10%	26.10%	0.73
	Apprenticeship	43.50%	52.20%	–
	GCE A‑levels	15.30%	13.00%	–
	University degree	14.10%	8.70%	–
Current or former smoker		52.40%	60.40%	0.36
Diabetes		22.40%	26.90%	0.54
Hyperlipidemia		50.60%	60.00%	0.29
Prior stroke		1.20%	1.90%	0.74
Prior MI		2.40%	0.00%	0.26
Heart failure		2.40%	0.00%	0.26
Coronary artery disease		3.50%	3.70%	0.96
Peripheral artery disease		1.20%	11.10%	<0.01
Cerebrovascular disease		2.40%	3.70%	0.22
COPD		4.70%	13.00%	0.08
CKD		1.20%	0.00%	0.42
Lipid lowering treatment		29.90%	43.10%	0.12
Antidiabetic treatment	Oral antidiabetics	16.50%	21.20%	0.73
	Insulin therapy	1.20%	1.90%	–
Antiplatelet drugs		18.20%	29.40%	0.14

*BMI* body mass index; *SBP* systolic blood pressure; *DBP* diastolic blood pressure; *MI* myocardial infarction; *COPD* chronic obstructive pulmonary disease; *CKD* chronic kidney disease

### Treatment

Antihypertensive treatment in the respective trial arms is shown in Table 2.

**Table 2:** Antihypertensive treatment in the respective study groups

		Standard	Intervention	*p*-value
Prior antihypertensive treatment		80.00%	81.10%	0.87
Number of different antihypertensive prescriptions	Prior to trial	1.7 ± 0.9	1.5 ± 0.8	0.17
	At trial start	1.8 ± 0.9	1.0 ± 0.2	<0.01
	At 6 months	2.0 ± 1.0	1.2 ± 0.5	<0.01
*Baseline*				
OLM/AML 20/5		1.2%	72.9%	–
OLM/AML 40/5		0.0%	2.1%	–
OLM/AML 40/10		0.0%	8.3%	–
OLM/AML/HCT 40/10/12.5		1.2%	4.2%	–
OLM/AML/HCT 40/10/25		2.5%	0.0%	–
Other OLM/AML/HCT combination^a^		8.6%	12.5%	–
*6 months*				
OLM/AML 20/5		1.2%	44.0%	–
OLM/AML 40/5		1.2%	14.0%	–
OLM/AML 40/10		3.5%	2.0%	–
OLM/AML/HCT 40/10/12.5		2.4%	14.0%	–
OLM/AML/HCT 40/10/25		1.2%	18.0%	–
Other OLM/AML/HCT combination^a^		7.2%	6.0%	–
SPC other than OLM/AML/HCT		38.8%	0.0%	<0.01
ACE inhibitors/ARB		45.9%	1.9%	<0.01
Beta-blockers		34.1%	5.7%	<0.01
CCB		27.1%	1.9%	<0.01
Diuretics		1.2%	0.0%	0.43
MRAs		2.4%	0.0%	0.26
Alpha-blockers		5.9%	1.9%	0.26
Other ^b^		9.4%	1.9%	0.08

*OLM* olmesartan; *AML* amlodipine; *HCT* hydrochlorothiazide; *SPC* single pill combination; *ACE* angiotensin converting enzyme; *ARB* angiotensin receptor blocker; *CCB* calcium channel blocker; *MRA* mineralocorticoid receptor antagonist

^a^Includes OLM/HCT single pill combinations and different dosing of the respective substances

^b^Includes alpha-agonists, other centrally acting agents, renin inhibitors and minoxidil

Prior to the trial enrolment, pretreated patients received an average of 1.6 ± 0.9 different antihypertensive prescriptions, which was similar between the standard of care and interventional arm (*p* = 0.17). Angiotensin-converting enzyme (ACE) inhibitors/angiotensin receptor blockers (52%), SPCs (41%) and beta-blockers (36%) were the most frequently used substance classes prior to enrolment. Since the use of the olmesartan/amlodipine/HCT study drug was not prohibited for practitioners in the standard care, 17% of patients enrolled in this trial arm received an SPC containing one of these substances. Other SPCs were used in 39% of patients, thus in total 56% of patients enrolled into the standard of care arm received an SPC at 6 months of follow-up. At 6 months, the number of different antihypertensive prescriptions was significantly lower in interventional care vs. standard care (1.2 ± 0.5 vs. 2.0 ± 1.0, *p* < 0.01).

### Office blood pressure reductions

#### Cluster level data

**Fig. 3:**
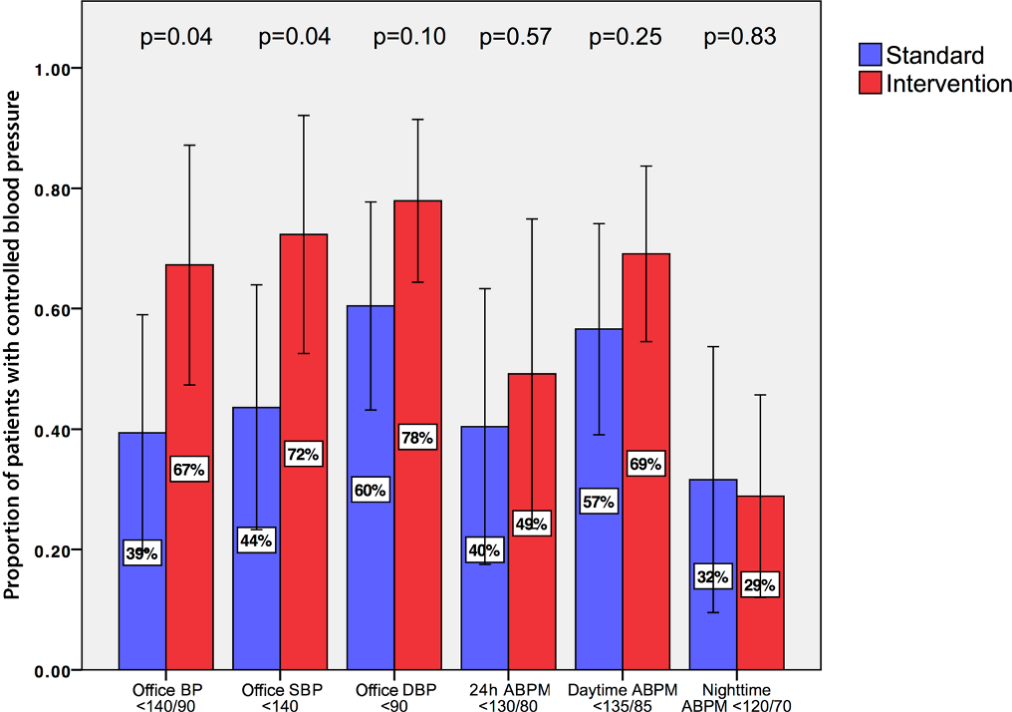
Graph showing the proportion of patients with controlled office blood pressure and ambulatory blood pressure levels at 6 months of follow-up. *p*-values are reported for differences between standard and interventional care. Additional data for control rates according to systolic and diastolic ambulatory blood pressure levels are presented in Supplementary Table 1. *BP* blood pressure, *SBP* systolic blood pressure, *DBP* diastolic blood pressure, *ABPM* ambulatory blood pressure measurement

Office BP was controlled in 52% ± 31% of patients after 6 months at a threshold of 140/90 mm Hg. At the cluster-level, 67% ± 26% of patients in interventional care and 39% ± 29% in standard care had their office BP controlled after 6 months of follow-up (Fig. 3). Accordingly, a significantly larger proportion of patients treated at sites which were randomized to interventional care vs. standard care achieved the office BP target (primary endpoint, mean between-group difference −27.9%, 95% CI −54.0%; −1.7%, *p* = 0.038, Fig. 3, Supplementary Table 1). An analysis that weighted the number of patients included at each site also showed significant improvements in favor of interventional care (Supplementary Table 1).

#### Patient level data

At 6 months, mean systolic/diastolic office BP was 135.9 ± 13.5/83.6 ± 9.3 mm Hg in interventional care and 142.4 ± 18.3/87.7 ± 11.5 mm Hg in standard care (Table 3).

**Table 3:** Office and ambulatory blood pressure levels at 6 months of follow-up with mean between-group differences

	Standard	Intervention	Mean difference	95% CI	*p*-value
Office SBP	142.38 ± 18.26	135.89 ± 13.53	−12.5	−18.8; −6.2	<0.01
Office DBP	87.68 ± 11.46	83.59 ± 9.28	−4.4	−8.6; −0.3	0.04
24 h SBP	130.65 ± 14.01	129.12 ± 13.21	−2.7	−7.9; 2.6	0.32
24 h DBP	79.34 ± 9.29	77.75 ± 9.28	−1.1	−4.1; 1.9	0.47
Daytime SBP	133.57 ± 14.8	131 ± 13.36	−2.7	−8.3; 3	0.35
Daytime DBP	81.65 ± 9.71	79.88 ± 9.47	−0.8	−4.1; 2.5	0.63
Nighttime SBP	122.85 ± 14.7	123.43 ± 15.51	−2.7	−8.4; 2.9	0.34
Nighttime DBP	72.76 ± 9.78	72.25 ± 10.47	−1.4	−5; 2.1	0.43

*SBP* systolic blood pressure, *DBP* diastolic blood pressure, *CI* confidence interval

**Fig. 4:**
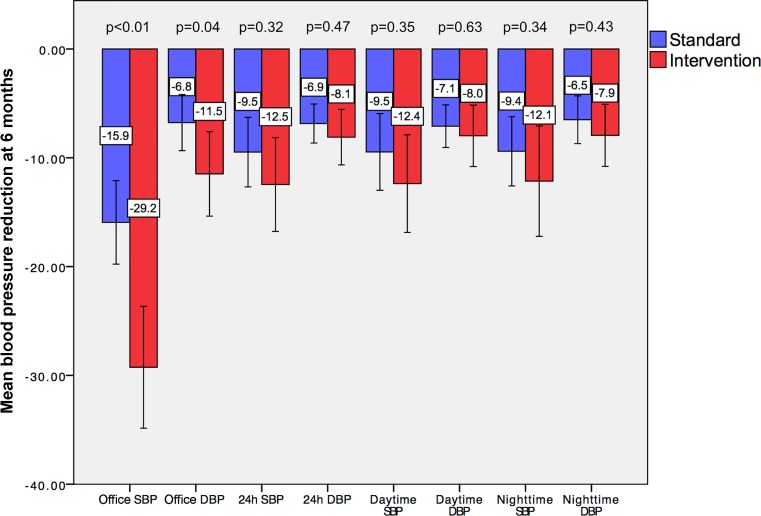
Mean office and ambulatory blood pressure reductions in standard and interventional care after 6 months of follow-up. *P*-values are reported for between-group differences. *SBP* systolic blood pressure, *DBP* diastolic blood pressure

Office BP reductions at the patient level were therefore greater in interventional vs. standard care (mean between-group difference −12.5 mm Hg, 95% CI −18.8; −6.2, *p* < 0.01 for office SBP and −4.4 mm Hg, 95% CI −8.6; −0.3, *p* = 0.04 for office DBP, Fig. 4).

### Ambulatory blood pressure reductions

#### Cluster level data

The 24h ABPM was controlled in 44% ± 33% of patients after 6 months at a threshold of 130/80 mm Hg. At the cluster level, 49% ± 33% of patients in interventional care and 40% ± 34% in standard care achieved the 24 h ABPM treatment target of 130/80 mm Hg (Fig. 3). The between-group difference was not statistically significant (mean between-group difference −8.8%, 95% CI −40.7%; 23.1%, *p* = 0.57, Supplementary Table 1). Daytime and nighttime ABPM reductions were also similar between groups in the unweighted analysis (Fig. 3). When weighting for cluster size, there was a significantly greater proportion of patients who achieved the daytime ABPM treatment target of 135/85 mm Hg in interventional vs. standard care (63% ± 14% vs. 49% ± 17%, mean between-group difference −13.6%, 95% CI −19.1%; −8.0%, *p* < 0.01, Supplementary Table 1).

#### Patient level data

At 6 months, mean systolic/diastolic 24 h ABPM was 129.1 ± 13.2/77.8 ± 9.28 mm Hg in interventional care and 130.7 ± 14.0/79.3 ± 9.3 mm Hg in standard care. Accordingly, 24 h ABPM reductions after 6 months of follow-up were similar in interventional vs. standard care (mean between-group difference −2.7 mm Hg, 95% CI −7.9; 2.6, *p* = 0.32 for 24 h SBP and −1.1 mm Hg, 95% CI −4.1; 1.9, *p* = 0.47 for 24 h DBP). Daytime and nighttime ambulatory BP at 6 months is presented in Table 3.

### The white coat effect in primary care

At baseline, systolic and diastolic office BP levels were significantly higher than the respective daytime ABPM values (mean difference 18.0 mm Hg, 95% CI 15.3; 20.7, *p* < 0.01 for systolic values and 6.6 mm Hg, 95% CI 4.7; 8.4, *p* < 0.01 for diastolic values). A similar, but less pronounced difference could be observed at 6 months (mean difference 7.6 mm Hg, 95% CI 5.2; 10.0, *p* < 0.01 for systolic values and 5.3 mm Hg, 95% CI 3.6; 7.1, *p* < 0.01 for diastolic values).

### Adverse events

Serious adverse events were infrequent and occurred at a similar rate between groups (interventional care 0 events, standard of care 4 events, *p* = 0.11). Of these 4 events, 2 were classified as potentially treatment related (one allergic reaction, one hypertensive urgency). Other adverse events such as fatigue, dizziness or leg edema occurred at a similar rate in the respective trial arms (Supplementary Table 2).

## Discussion

The main findings of our study are:

1. In both trial arms, many patients with previously elevated office BP could be easily controlled with a relatively low medication burden when included into a trial dedicated to improve BP control.
2. A significantly higher proportion of patients in interventional vs. standard care had their office BP controlled after 6 months of follow-up.
3. Interventional and standard care were similar regarding the improvement in the ABPM profile.
4. BP reductions were achieved with a significantly lower medication burden in interventional vs. standard care.

Accordingly, an overall clinical benefit with the prespecified titration regimen was observed using a SPC, a strategy that could be easily adopted in a primary care setting. The use of SPCs and simplification of treatment regimens have been found to improve adherence, which might translate into a sustained BP lowering effect [11, 12]. In Austria it could recently be shown that only 41% of diagnosed, treated and predominantly adherent patients, who actively approached a pharmacy to obtain the antihypertensive medication have controlled BP levels [4]. This previous study, and also the present trial suggest that poor BP control is more due to low adherence and the lack of adequate therapy intensification (i.e. physician’s inertia) than treatment resistance. On average, patients were pretreated with 1–2 different antihypertensive drugs, leaving sufficient room for therapy intensification. To overcome these barriers, disease management programs addressing both patient and physician-related factors, such as the Canadian Hypertension Education Program (CHEP) or the Austrian herz.leben program seem worthwhile to improve BP control and reduce stroke-related morbidity and mortality [13–16].

The STITCH trial randomized 45 family practices in Canada to standard care vs. a simplified treatment algorithm with step-wise uptitration of antihypertensive therapy. Corresponding to the results of the present study, 65% in interventional care and 53% in standard care had their BP controlled after 6 months [8]. These observations were based on office BP readings. As this and other studies show, contemporary trials should incorporate home BP readings, unattended automated office BP or ABPM to provide accurate results [17–20]. Although the majority of patients in this study were pretreated with antihypertensive drugs (most likely by the same GP who was responsible for enrolment into the trial), there was still a decline in the white-coat effect over time.

Based on these data contemporary disease management programs might primarily address 1) the improvement of adherence by simplification of treatment regimens, 2) physician’s inertia, and 3) a widespread adoption of automated office BP or ABPM with the support of healthcare providers [13, 21].

### Strengths and limitations

Compared to previous trials, 96% of eligible patients underwent ABPM at baseline and after 6 months follow-up. This strengthens our results, since office BP values have been shown to be insufficient to judge treatment effects in HTN trials [20, 22, 23]. Due to slow recruitment, which led to the premature termination of the trial, the analysis lacks sufficient statistical power and can only be regarded as hypothesis generating.

## Conclusion

In both groups statistically and clinically significant BP reductions were observed after 6 months. In the interventional care group, a larger proportion of patients achieved the office BP target compared to standard care. The 24 h ambulatory blood pressure levels were controlled in 44% of patients at 6 months, without significant differences between groups. The respective BP reductions were achieved with a significantly lower medication burden in interventional care.

## Caption Electronic Supplementary Material

Additional data for primary and secondary outcomes, and the incidence of adverse events
