# Supplementary material for: Lowering blood pressure in primary care in Vienna (LOW-BP-VIENNA): A cluster-randomized trial
Source: Wien Klin Wochenschr. 2018 Aug 15;130(23):698–706. doi: 10.1007/s00508-018-1374-4 (PMC6290730; doi:10.1007/s00508-018-1374-4)
Supplement: Supplementary file 1 — Additional data for primary and secondary outcomes, and the incidence of adverse events [file 508_2018_1374_MOESM1_ESM.doc]

**Supplementary Table 1**: Primary and secondary outcomes (proportion of patients with controlled blood pressure) at the cluster level

| **Unweighted Analysis** |  |  |  |  |  |
| --- | --- | --- | --- | --- | --- |
|  | **Standard** | **Intervention** | **Mean difference** | **95% CI** | **p-value** |
| Office BP <140/90 | 39% ± 29% | 67% ± 26% | -27.9% | -54.% ; -1.7% | 0.04 |
| Office SBP <140 | 44% ± 30% | 72% ± 26% | -28.7% | -55.5% ; -1.9% | 0.04 |
| Office DBP <90 | 60% ± 26% | 78% ± 18% | -17.5% | -38.7% ; 3.8% | 0.10 |
| ABPM 24h <130/80 | 40% ± 34% | 49% ± 33% | -8.8% | -40.7% ; 23.1% | 0.57 |
| ABPM 24h SBP <130 | 52% ± 32% | 58% ± 31% | -6.1% | -35.9% ; 23.6% | 0.67 |
| ABPM 24h DBP <80 | 55% ± 34% | 62% ± 25% | -6.5% | -35.2% ; 22.1% | 0.64 |
| ABPM Day <135/85 | 57% ± 26% | 69% ± 19% | -12.5% | -34.4% ; 9.4% | 0.25 |
| ABPM Day SBP <135 | 59% ± 26% | 71% ± 20% | -11.2% | -33.5% ; 11.% | 0.30 |
| ABPM Day DBP <85 | 75% ± 18% | 79% ± 18% | -4.2% | -21.1% ; 12.8% | 0.61 |
| ABPM Night <120/70 | 32% ± 33% | 29% ± 22% | 2.7% | -24.2% ; 29.7% | 0.83 |
| ABPM Night SBP <120 | 40% ± 32% | 42% ± 31% | -1.8% | -31.5% ; 28.% | 0.90 |
| ABPM Night DBP <70 | 39% ± 31% | 43% ± 26% | -3.5% | -30.7% ; 23.8% | 0.79 |
|  |  |  |  |  |  |
| **Weighted Analysis** |  |  |  |  |  |
|  | **Standard** | **Intervention** | **Mean difference** | **95% CI** | **p-value** |
| Office BP <140/90 | 39% ± 20% | 61% ± 21% | -22.3% | -29.4% ; -15.2% | <0.01 |
| Office SBP <140 | 47% ± 23% | 67% ± 21% | -19.6% | -27.4% ; -11.9% | <0.01 |
| Office DBP <90 | 55% ± 20% | 76% ± 15% | -20.6% | -26.8% ; -14.4% | <0.01 |
| ABPM 24h <130/80 | 34% ± 20% | 37% ± 28% | -2.9% | -11.% ; 5.1% | 0.47 |
| ABPM 24h SBP <130 | 46% ± 22% | 48% ± 27% | -2.3% | -10.6% ; 6.1% | 0.59 |
| ABPM 24h DBP <80 | 48% ± 22% | 56% ± 18% | -7.3% | -14.5% ; -.2% | 0.04 |
| ABPM Day <135/85 | 49% ± 17% | 63% ± 14% | -13.6% | -19.1% ; -8.% | <0.01 |
| ABPM Day SBP <135 | 55% ± 19% | 65% ± 16% | -9.5% | -15.6% ; -3.4% | <0.01 |
| ABPM Day DBP <85 | 66% ± 14% | 74% ± 12% | -8.2% | -12.7% ; -3.7% | <0.01 |
| ABPM Night <120/70 | 29% ± 20% | 30% ± 20% | -0.2% | -7.2% ; 6.8% | 0.95 |
| ABPM Night SBP <120 | 41% ± 22% | 41% ± 29% | 0.4% | -8.1% ; 9.% | 0.92 |
| ABPM Night DBP <70 | 34% ± 18% | 37% ± 18% | -2.9% | -9.1% ; 3.3% | 0.35 |

Legend: SBP: systolic blood pressure; DBP: diastolic blood pressure; ABPM: ambulatory blood pressure measurement

**Supplementary Table 2**: Incidence of adverse events in the respective study groups

|  | Standard | Intervention | p-value |
| --- | --- | --- | --- |
| Serious adverse events | 4 | 0 | 0.11 |
| Dizzines | 4 | 5 | NS |
| Leg edema | 2 | 2 | NS |
| Fatigue | 3 | 3 | NS |
| Palpitations | 2 | 1 | NS |
| Xerostomia | 0 | 1 | NS |
| Paresthesia | 1 | 1 | NS |
| Sleep disorder | 0 | 1 | NS |
| Headache | 2 | 1 | NS |
| Gastrointestinal disorders | 0 | 1 | NS |
